# MASP1 modulation as a novel therapeutic target in severe pediatric pertussis: insights from a multi-omics approach

**DOI:** 10.1128/iai.00271-24

**Published:** 2025-01-22

**Authors:** Lin Xu, Caiying Wang, Yuhuan Liu, Yanlan Zhang, Zhen Li, Lin Pang

**Affiliations:** 1 Department of Pediatrics, Beijing Ditan Hospital Affiliated to Capital Medical University 12638 , Beijing, China; 2 Beijing Chaoyang District Center for Disease Control and Prevention 661283 , Beijing, China; University of California Davis, Davis, California, USA

**Keywords:** pertussis, MASP1 protein, microbial diversity, transcriptome analysis, machine learning, infection model

## Abstract

Pertussis, a severe infectious disease in children, has become increasingly prominent in recent years. This study aims to investigate the role of the MASP1 protein in severe pertussis in children through multi-omics analysis, providing a theoretical basis for the development of novel therapeutic strategies. The study retrieved macro-genome and 16S rRNA data of pediatric pertussis from public databases to analyze microbial diversity and specific flora abundance, conducting pathway functional enrichment analysis. Differential expression analysis of transcriptome data and Gene Ontology (GO)/Kyoto Encyclopedia of Genes and Genomes (KEGG) functional enrichment analysis, combined with machine learning, identified the key gene MASP1. A *Bordetella pertussis* infection model was established using human bronchial epithelial cell line HBE135-E6E7 to validate MASP1 expression changes and investigate its relationship with airway epithelial cell damage by constructing cell lines overexpressing and knocking down MASP1. Finally, the impact of inhibiting MASP1 expression on infection symptoms was evaluated using a mouse pertussis infection model. The results revealed significant differences in microbial diversity and specific flora abundance between healthy children and those with pertussis, with MASP1 significantly upregulated in severe pertussis and its inhibition alleviating infection symptoms. The study highlights the critical role of MASP1 in pertussis, providing a crucial foundation for developing therapeutic strategies targeting MASP1.

## INTRODUCTION

Pertussis, caused by *Bordetella pertussis*, is a highly contagious respiratory disease primarily affecting children (1, 2). Despite the widespread promotion of pertussis vaccination globally, there has been a recent increase in the incidence of the disease in certain regions, particularly affecting infants and children who are not fully immunized (3). Clinical symptoms of pertussis include persistent severe coughing, breathing difficulties, and potential complications, such as pneumonia and encephalopathy, placing significant strain on patients’ families and the public healthcare system (4–6). Conventional antibiotic therapy has some efficacy against pertussis; however, its clinical effectiveness is limited due to antibiotic resistance issues (7–9). Consequently, in-depth exploration of the pathogenic mechanisms of pertussis and the search for new therapeutic targets have emerged as crucial research areas in need of immediate attention (10, 11).

Currently, research on pertussis predominantly focuses on understanding the pathogenic mechanisms of the pathogen and improving vaccines (12). The application of multi-omics technologies, such as macro-genomics and transcriptomics, enables scientists to comprehensively comprehend the interactions between the pertussis pathogen and the host (13–15). The 16S rRNA analysis technology plays a significant role in revealing the structure and diversity of nasopharyngeal microbiota, offering a new perspective for the pathological study of pertussis. However, existing research tends to be descriptive, lacking in-de–pth exploration of the specific functions of key genes and their regulatory mechanisms during pertussis infection (16, 17). For instance, although it is known that certain genes exhibit abnormal expression during infection, further experimental data are needed to understand how they affect disease progression and if they could serve as potential therapeutic targets (18, 19).

Mannan-binding lectin serine protease 1 (MASP1) is a serine protease in the complement system that plays a crucial role in innate immune responses and inflammation (20). In recent years, an increasing number of studies have identified various expression patterns of MASP1 in infectious diseases, suggesting its potential key role in pathogen infection processes (21, 22). However, the specific function and regulatory mechanisms of MASP1 in pertussis remain unclear. Through machine learning algorithms and protein–protein interaction (PPI) network analysis, this study identified MASP1 as a characteristic gene of pertussis, revealing its significantly upregulated expression in pertussis patients. This finding suggests that MASP1 may play a crucial role in the pathological process of pertussis infection, warranting further in-depth investigation.

This study employed a multi-omics analysis approach, starting with macro-genome and 16S rRNA data to analyze the microbial diversity and specific bacterial abundance in pediatric pertussis patients compared with a healthy control group (23–25). Differential gene expression and functional enrichment analyses were conducted using transcriptomic data, identifying the key gene MASP1. Subsequently, the expression changes of MASP1 were validated on the HBE135-E6E7 human bronchial epithelial cell line *in vitro*, and the relationship between MASP1 and pertussis-induced airway epithelial cell damage was investigated by constructing MASP1-overexpressing and knockdown cell lines. Finally, the impact of inhibiting MASP1 on infection symptoms was evaluated using a mouse model of pertussis infection. Through this comprehensive approach, the study aims to systematically elucidate the role and mechanisms of MASP1 in the process of pertussis infection (26, 27).

The main objective of this study is to investigate the role of the MASP1 protein in severe pertussis in children through multi-omics analysis, with the aim of providing a theoretical basis for the development of novel therapeutic strategies for this disease. Initially, the study identified significant differences in the nasal-pharyngeal microbial diversity and specific bacterial abundance between the healthy pediatric control group and pertussis pediatric patient group, laying the foundation for further exploration of the role of the microbiota in the pathogenesis of pertussis. Subsequently, through protein–protein interaction network analysis and machine learning algorithms, the study screened and validated the expression changes of the characteristic pertussis gene MASP1, revealing its crucial role in the process of pertussis infection. *In vitro* experiments and *in vivo* animal model experiments further confirmed that inhibiting MASP1 expression can significantly alleviate pertussis infection symptoms. These findings not only deepen our understanding of the pathogenic mechanisms of pertussis but also provide important scientific evidence for the future development of precision treatment strategies targeting MASP1 in clinical practice. In summary, this study holds significant scientific significance and potential application value in the pathological research and clinical treatment of pertussis.

## RESULTS

### The impact of childhood pertussis on nasopharyngeal microbial diversity

Pertussis, an acute respiratory infection caused by *B. pertussis*, poses a significant threat to children’s health (28). Despite a significant reduction in pertussis incidence due to vaccination programs in recent years, cases of pertussis continue to occur globally (28). The nasopharynx serves as the primary gateway for respiratory infections, and the composition and diversity of its microbiota play a crucial role in maintaining the host’s health.

**Fig 1:**
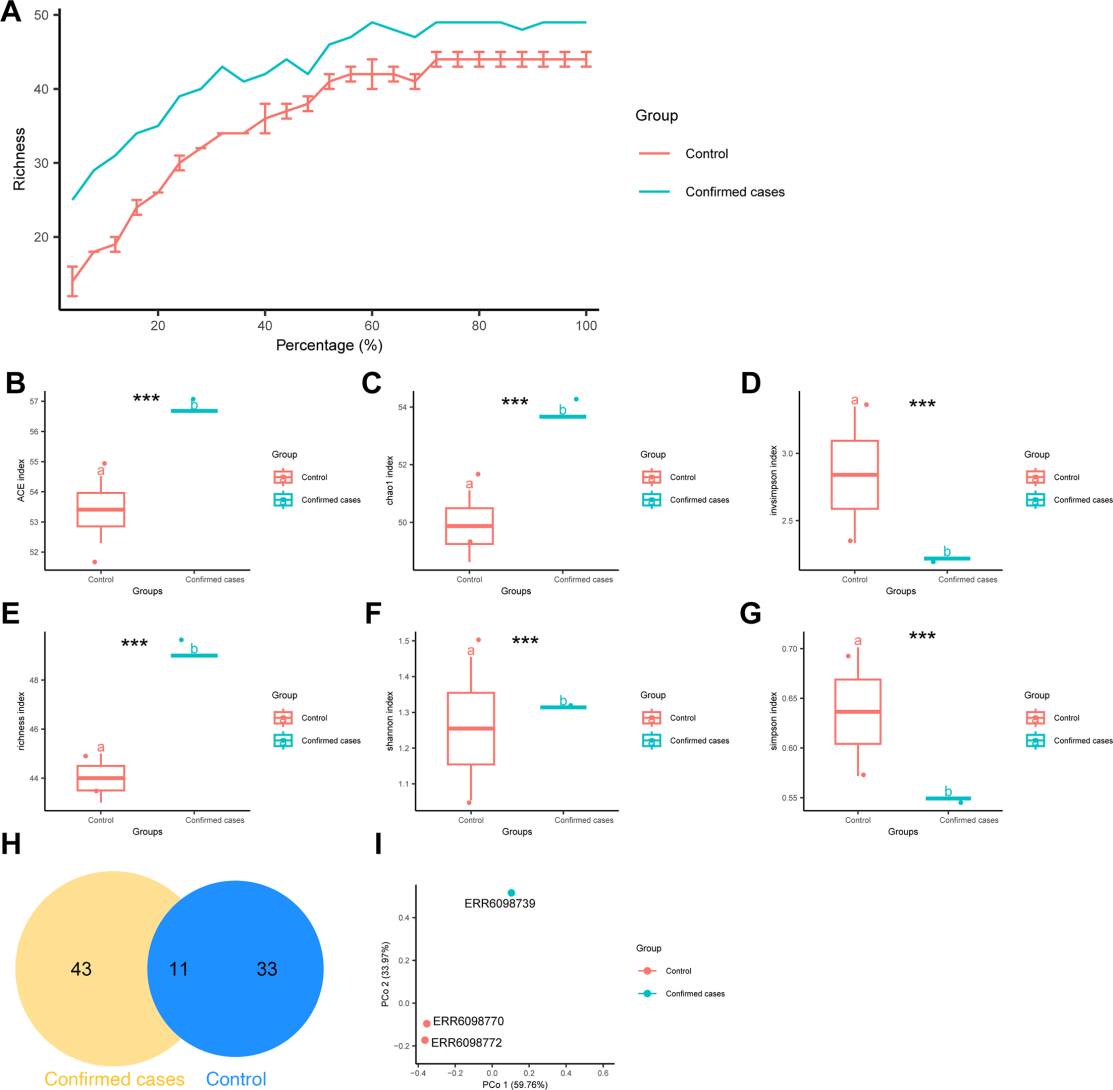
Comparison of nasopharyngeal flora species diversity between Confirmed cases and Control groups. (**A**) Rarefaction curve of nasopharyngeal flora alpha diversity in Confirmed cases (*n* = 12) and Control (*n* = 9) samples; (**B-G**) Alpha diversity analysis of nasopharyngeal flora in Confirmed cases (*n* = 12) and Control (*n* = 9) samples using ACE, Chao1, Invsimpson, Richness, Shannon, and Simpson indices. The notation “^ns^” signifies that the difference is not significant when *P* > 0.05, whereas “***” indicates a statistically significant difference when *P* < 0.001; (**H**) Venn diagram of amplicon sequence variants in nasopharyngeal flora of Confirmed cases (*n* = 12) and Control (*n* = 9) samples; (**I**) PCoA of nasopharyngeal flora in Confirmed cases (*n* = 12) and Control (*n* = 9) samples.

To further investigate the impact of pertussis on the nasopharyngeal microbiota, we retrieved 16S RNA data from the EMBL-EBI database (project PRJEB45720), which included nasopharyngeal samples from nine healthy children in the control group (Control) and 12 children with confirmed pertussis (Confirmed cases). Data from children who had used antibiotics were excluded to avoid interference with microbiota composition. Alpha rarefaction curve analysis showed that as the sample size percentage increased, the richness in both the Control and Confirmed cases groups gradually slowed and eventually reached saturation. Additionally, nasopharyngeal microbiota diversity was significantly higher in the Confirmed cases group than in the Control group (Fig. 1A).

Alpha diversity serves as an indicator of species composition within samples, considering both the quantity and abundance dimensions. Commonly used calculation methods include abundance-based coverage estimator (ACE), Chao1, InvSimpson, Richness, Shannon, and Simpson indices. The results indicated that the Simpson and InvSimpson indices were notably higher in the Control group compared with the Confirmed cases group, while the ACE, Chao1, InvSimpson, and Richness indices were significantly higher in the Confirmed cases group relative to the Control group, suggesting that pertussis may potentially impact the diversity and composition of nasopharyngeal flora (Fig. 1B through G).

Subsequently, we conducted a Venn analysis on the amplicon sequence variants (ASVs) enriched in the Control and Confirmed cases group, revealing 33 ASVs unique to the Confirmed cases group compared with the Control group (Fig. 1H). PCoA demonstrated a distinct separation between samples from the two groups (Fig. 1I). Disparities in the abundance of nasopharyngeal flora between the Control and Confirmed cases groups were computed using the R script comparison. R, heatmaps, and Manhattan plots were generated (Fig. S1). The results indicated significant differences in ASVs at the class level within the bacterial phylum (Bacteria) between the Confirmed cases and Control groups. Specifically, compared with the Confirmed cases group, the Control group exhibited 24 significantly depleted ASVs and 23 significantly enriched ASVs.

In conclusion, pertussis in children has been shown to impact nasopharyngeal microbial diversity.

### The impact of treatment on the composition of nasopharyngeal flora

**Fig 2:**
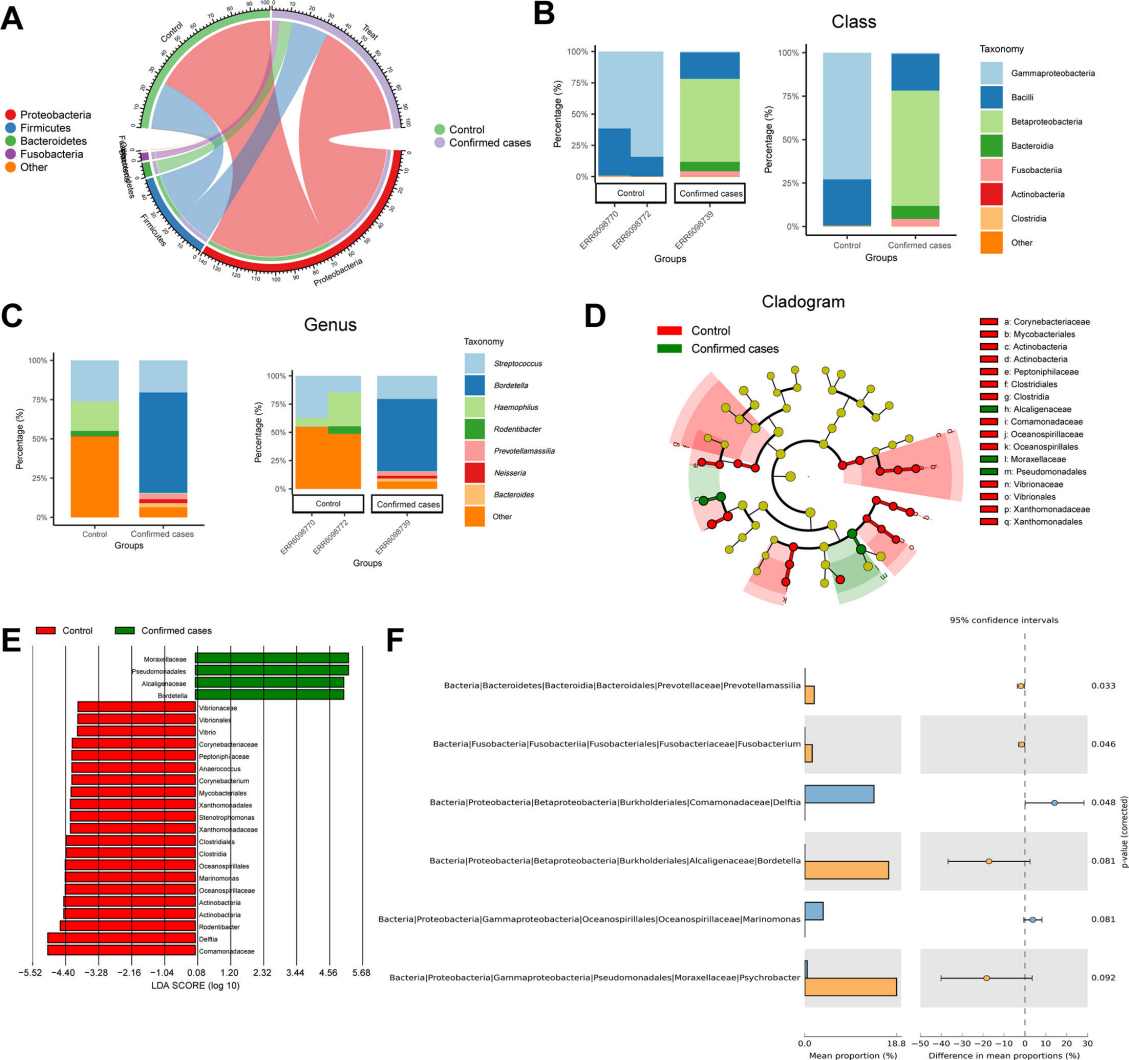
Analysis of species composition differences in nasopharyngeal flora between the Control and Confirmed cases group at different levels. (**A**) Circular plot of species abundance of nasopharyngeal flora in the Control and Confirmed cases group at the phylum level; (**B-C**) Stack bar charts of species abundance of nasopharyngeal flora in the Control and Confirmed cases group at the class and genus levels, respectively (the left side shows the stack bar charts of mean species abundance of nasopharyngeal flora, and the right side displays the stack bar charts of species abundance combining sample grouping); (**D**) Cladogram of species abundance in nasopharyngeal flora of the Control and Confirmed cases group, where the circles radiating from the inside out represent taxonomic levels from phylum to genus, with circle size indicating relative abundance, yellow nodes indicating species with no significant difference, green nodes representing microbial taxa with higher abundance in the Confirmed cases group, and red nodes representing microbial taxa with higher abundance in the Control group; (**E**) LDA value distribution bar chart of species abundance in nasopharyngeal flora of the Control and Confirmed cases group; (**F**) Extended error bar plot of species abundance in nasopharyngeal flora of the Control and Confirmed cases group at the genus level (blue bars indicate microbial taxa with higher abundance in the Control group, while orange bars indicate microbial taxa with higher abundance in the Confirmed cases group); Control group (*n* = 9), Confirmed cases group (*n* = 12).

Further investigation was conducted on the species composition by analyzing the distribution of species in the Confirmed cases and Control groups at the phylum, class, order, family, and genus levels using pie charts and stacked bar graphs (Fig. 2A and B; Fig. S2). The results revealed that at the phylum level, the predominant bacterial strains in both groups were Proteobacteria, Firmicutes, Bacteroidetes, and Fusobacteria. However, the Confirmed cases group exhibited a significantly higher abundance of Bacteroidetes and Fusobacteria at the phylum level compared with the Control group (Fig. 2A; Fig. S2A). At the class level, Betaproteobacteria, Bacteroidia, and Fusobacteriia were notably more abundant in the Confirmed cases group than in the Control group, while the proportion of Gammaproteobacteria decreased (Fig. 2B). Moreover, at the genus level, *Haemophilus* was more prevalent in the Control group, whereas *Bordetella*, *Prevotellamassilia*, *Neisseria*, and *Bacteroides* were more abundant in the Confirmed cases group (Fig. 2C).

In order to investigate the specific differences in species abundance of nasopharyngeal flora between the Confirmed cases and Control groups, we performed LEfSe analysis and visualized the results. The LEfSe analysis indicated that in the nasopharyngeal samples of the Confirmed cases group, the relative abundance of Moraxellaceae, Pseudomonadales, Alcaligenaceae, and Bordetella was significantly higher compared with the Control group. On the other hand, Vibrionaceae_Vibrionales_Vibrio, Clostridiales_Clostridia, Xanthomonadaceae_Xanthomonadales, and Actinobacteria were more abundant in the Control samples (Fig. 2D and E). Further visualization of the species composition of nasopharyngeal flora was conducted using STAMP software, revealing that in the Confirmed cases group, the relative abundance of Prevotellaceae_Prevotellamassilia, Fusobacteriaceae_Fusobacterium, and Alcaligenaceae_Bordetella was higher compared with the Control group (Fig. 2F).

To investigate the differences in the functional composition of nasopharyngeal flora between the Confirmed cases and Control groups, we utilized the PICRUSt2 software based on the abundance data at the genus level for KEGG enrichment analysis and visualized the results using STAMP software. The analysis revealed significant differences in the functional profiles of nasopharyngeal flora between the Confirmed cases and Control groups (*P* < 0.05), with a total enrichment of 46 signaling pathways (Fig. S3). Among these pathways are signal transduction mechanisms, oxidative phosphorylation, ether lipid metabolism, pantothenate and CoA biosynthesis, citrate cycle (TCA cycle), glycerolipid metabolism, protein processing in the endoplasmic reticulum, carbon fixation pathways in prokaryotes, toluene degradation, and Parkinson’s disease were among the top 10 pathways (Fig. S4A), with signal transduction mechanisms ranking first. According to the literature, *B. pertussis* activates host cell signaling pathways through toxins and surface molecules crucial for pathogen adhesion, invasion, and immune evasion strategies (29), aligning with the trends observed in our analysis (Fig. S4B). In conclusion, the variation in nasopharyngeal flora may enhance pertussis through signal transduction mechanisms. Therefore, there are significant differences in the composition of the nasopharyngeal microbiota between the Confirmed cases and Control groups, suggesting that these microbiota differences are of important significance in understanding the pathogenesis of pertussis.

### Identifying key factors in pertussis through transcriptome sequencing: MASP1

**Fig 3:**
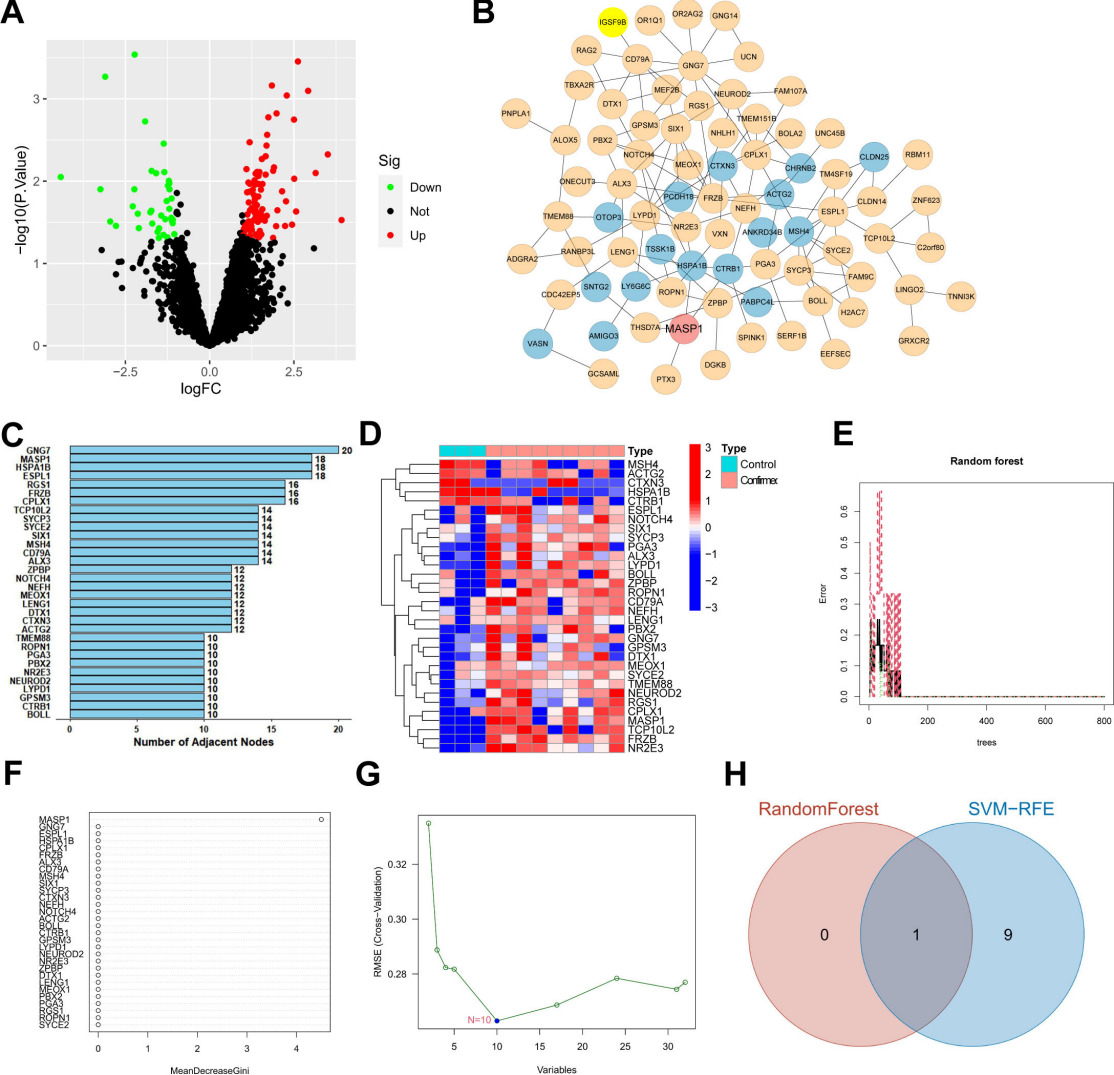
Machine learning algorithm for screening pertussis-related mRNA. (**A**) Volcano plot of differential analysis between three normal human airway epithelial cell samples and nine *B. pertussis*-stimulated human airway epithelial cell samples in the GSE182807 data set; (**B**) Network diagram of candidate target genes interactions, where each circle represents a gene, lines between circles indicate gene interactions, blue circles indicate downregulated genes, and red and orange circles indicate upregulated genes; (**C**) Statistics of core gene adjacency nodes in the gene interaction network, with the x-axis representing the adjacency node values and the y-axis representing gene names; (**D**) Expression profiles of genes with adjacency nodes greater than eight in the sequencing data, where Control represents normal airway epithelial cell samples, and Confirmed cases group represents *B. pertussis*-stimulated human airway epithelial cell samples; (**E-F**) Results of RF algorithm; (**G**) Analysis results from SVM-RFE; (**H**) Venn diagram showing the intersection of pertussis-related mRNA selected by RF algorithm and SVM-RFE algorithm.

To further identify genes closely associated with pertussis, we retrieved the pertussis-related data set GSE182807 from the GEO database. This data set comprises three samples of normal human airway epithelial cells and nine samples of human airway epithelial cells stimulated by *B. pertussis*. From these samples, we identified a total of 129 DEGs, consisting of 91 upregulated genes and 38 downregulated genes (Fig. 3A). Subsequently, we performed GO and KEGG analyses on these DEGs. The analysis revealed that these genes are primarily involved in biological processes, such as B cell proliferation, cell–cell adhesion through plasma-membrane adhesion molecules, and cell differentiation. The KEGG enrichment analysis indicated that the DEGs are mainly enriched in signaling pathways, including primary immunodeficiency, cell adhesion molecules (CAMs), and antigen processing and presentation (Fig. S5). These findings suggest a potential role for these genes in immune responses, cell adhesion, and pathogen invasion.

We performed an interaction analysis on the 129 DEGs and constructed a gene interaction network (Fig. 3B). Upon calculating the number of adjacent nodes for each gene in the network, we found that only 32 genes had more than eight adjacent nodes (Fig. 3C). The expression levels of these 32 genes are depicted in Fig. 3D. Furthermore, we assessed the importance of these 32 genes using the RF algorithm (Fig. 3E and F) and identified pertussis feature genes through the SVM-RFE analysis method (Fig. 3G), pinpointing a key mRNA: MASP1 (Fig. 3H). Prior studies have indicated that MASP1 plays a role in exacerbating respiratory infections (30, 31).

In summary, our screening has identified MASP1 as a characteristic gene of pertussis. The expression level of this gene may be associated with the susceptibility and severity of pertussis in children.

### Overexpression of MASP1 aggravates *B. pertussis*-induced airway epithelial cell damage

**Fig 4:**
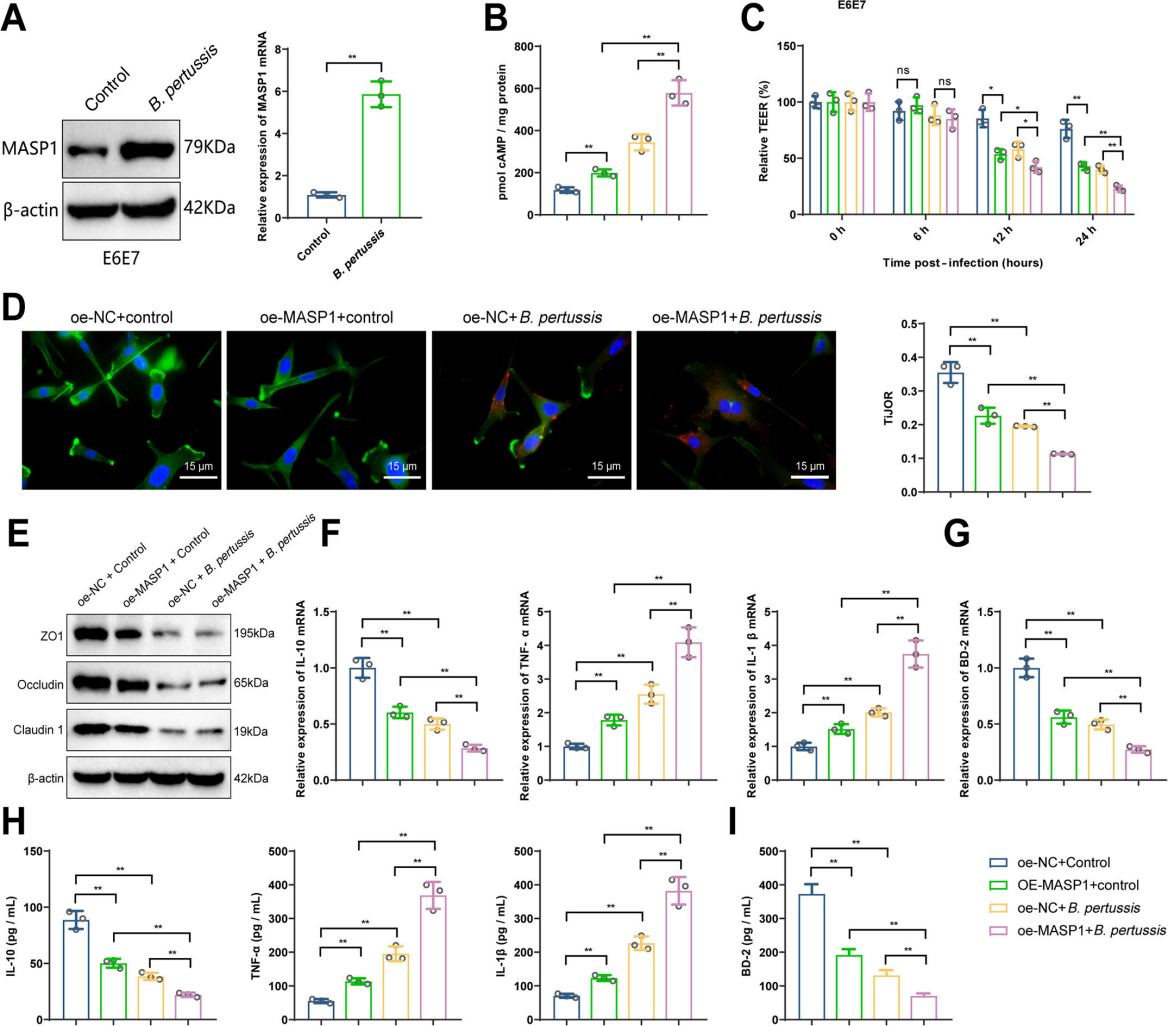
Impact of MASP1 overexpression on severe damage induced by *B. pertussis* in HBE135-E6E7 cells. (**A**) Detection of MASP1 expression changes in HBE135-E6E7 cells at 24 h post-*B. pertussis* infection by RT-qPCR and Western blot; (**B**) Measurement of cAMP levels in oe-NC + Control, oe-NC + *B. pertussis*, oe-MASP1 + Control, and oe-MASP1 + *B. pertussis* groups; (**C**) TEER percentage at different time points (0, 6, 12, and 24 h) in each group; (**D**) Representative images of tight junction detection by immunofluorescence (left) and statistical analysis of tight junction tissue integrity (right) (scale bar = 15 µm); (**E**) Expression of tight junction-associated markers in each group detected by Western blot; (**F-I**) Expression and secretion of inflammatory cytokines and antimicrobial peptide BD-2 in each group measured by RT-qPCR and ELISA; ^ns^*P* >0.05, *P < 0.05, **P < 0.01; Cell experiments were conducted in triplicate.

To investigate the relationship between MASP1 and the severity of pertussis, we used the human airway epithelial cell line HBE135-E6E7 (E6E7) to establish a *B. pertussis* infection model, as described in the Methods section, and measured intracellular cAMP levels (32). Results showed that after 24 h of infection, intracellular cAMP levels in the infected group (*B. pertussis*) were significantly elevated compared with the uninfected control group (Control) (Fig. S6A). We then examined MASP1 expression in HBE135-E6E7 cells, and the results indicated that MASP1 expression at both mRNA and protein levels was significantly upregulated in the *B. pertussis* group compared with the Control group (Fig. 4A), suggesting that MASP1 may be involved in the *B. pertussis* infection process.

To further analyze the impact of MASP1 on *B. pertussis*-induced airway epithelial cell damage, we generated MASP1 overexpression and knockdown cell lines in HBE135-E6E7 cells, along with their respective controls (designated as oe-MASP1, oe-NC, sh-MASP1, and sh-NC) (Fig. S6B and C), and performed *B. pertussis* infection treatments (24 h), dividing the samples into Control and *B. pertussis* groups. First, we measured changes in intracellular cAMP levels. Results showed that cAMP levels were significantly elevated in the oe-MASP1 + Control group compared with the control group (oe-NC + Control). Furthermore, cAMP levels were significantly higher in the *B. pertussis* infection group (oe-NC + *B. pertussis*) compared with the control group (oe-NC + Control), and cAMP levels were also significantly elevated in the oe-MASP1 + *B. pertussis* group compared with the oe-MASP1 + Control group (Fig. 4B).

Transepithelial electrical resistance (TEER), an important indicator of epithelial function (33), was measured, and results showed that after 12 and 24 h of infection, TEER was significantly decreased in the oe-MASP1 + Control group compared with the control group (oe-NC + Control). In addition, TEER was significantly reduced in the oe-NC + *B. pertussis* group compared with the control group (oe-NC + Control), and the oe-MASP1 + *B. pertussis* group showed an even greater reduction in TEER compared with the oe-MASP1 + Control group (Fig. 4C). The tight junction organization rate (TiJOR) also showed a significant decrease after 24 h of infection (Fig. 4D). Analysis of tight junction-related proteins indicated that, under infection conditions, the expression of ZO1, Occludin, and Claudin 1 was significantly lower in the oe-MASP1 + Control group compared with oe-NC + *B. pertussis*, and similarly, these proteins were markedly reduced in the oe-MASP1 + *B. pertussis* group compared with oe-NC + *B. pertussis* (Fig. 4E), suggesting that MASP1 expression promotes *B. pertussis*-induced disruption of epithelial tight junction integrity.

Measurements of inflammatory cytokines and antimicrobial peptides showed that, under infection conditions, TNF-α and IL-1β expression levels were upregulated in the oe-MASP1 + *B. pertussis* group compared with oe-NC + *B. pertussis*, while IL-10 and BD-2 levels were significantly downregulated (Fig. 4F through I), indicating that MASP1 expression enhances the inflammatory response induced by *B. pertussis* and further suppresses antimicrobial peptide expression. In contrast, results from the sh-NC + *B. pertussis* and sh-MASP1 + *B. pertussis* groups demonstrated that MASP1 inhibition alleviated *B. pertussis*-induced epithelial cell damage. Specifically, cAMP levels were lower, tight junction integrity was better, inflammation was suppressed, and BD-2 expression was higher in the sh-NC + Control group compared with the sh-NC + *B. pertussis* group. Additionally, the sh-MASP1 + *B. pertussis* group had lower cAMP levels, improved tight junction integrity, reduced inflammation, and higher BD-2 expression compared with the sh-NC + *B. pertussis* group (Fig. S7A through H), further indicating that MASP1 plays an important role in airway epithelial cells during *B. pertussis* infection.

These results suggest that MASP1 expression is upregulated in HBE135-E6E7 cells following *B. pertussis* infection, and overexpression of MASP1 exacerbates *B. pertussis*-induced cell damage in HBE135-E6E7 cells.

### Inhibition of MASP1 alleviates infection symptoms caused by *B. pertussis*

**Fig 5:**
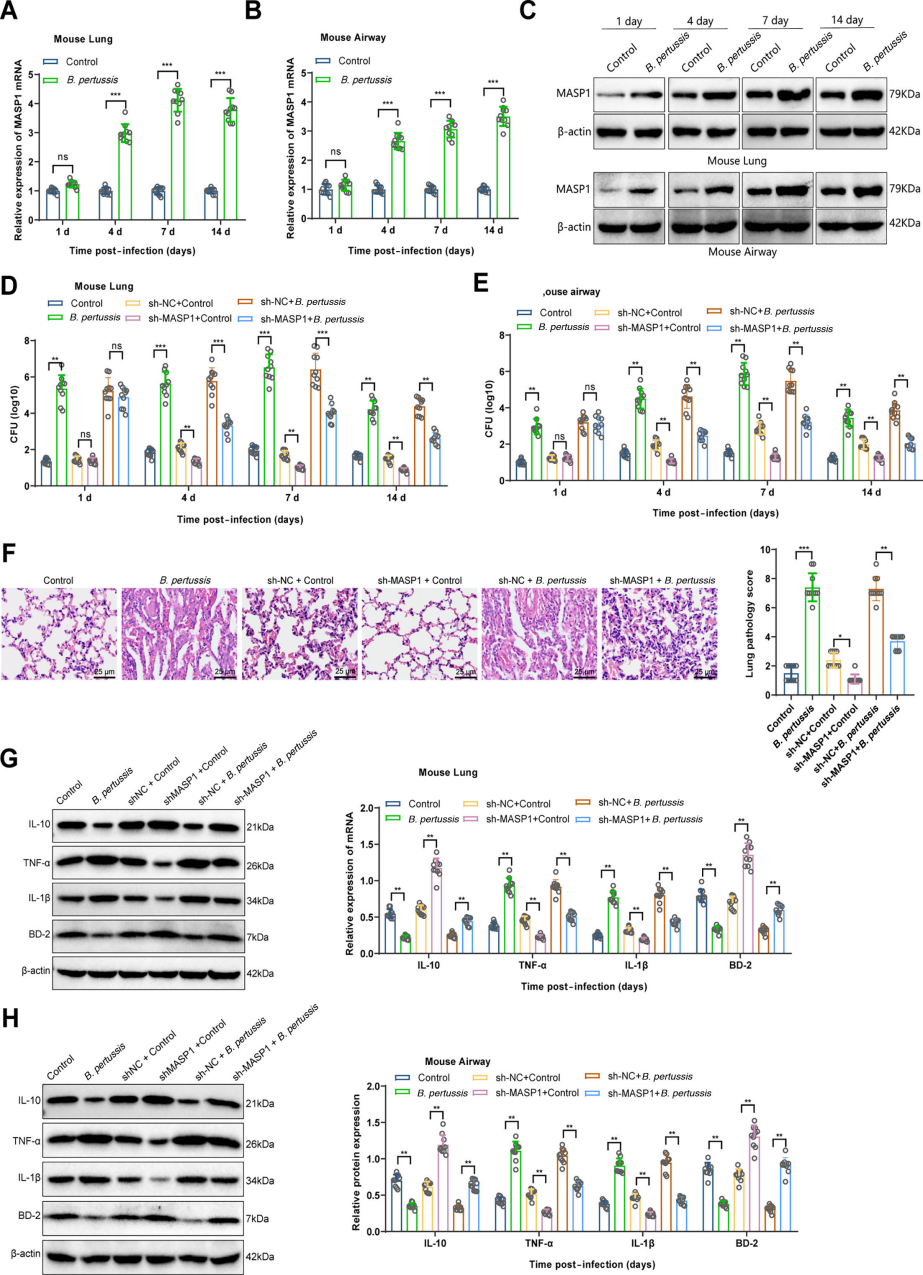
Effects of MASP1 inhibition on clinical symptoms and lung lesions in mice infected with *B. pertussis*. Note: (**A-C**) Expression of MASP1 in lung and airway tissues of mice at different time points (1, 4, 7, and 14 days) post-*B. pertussis* infection detected by RT-qPCR and Western blot; (**D-E**) Bacterial burden in lung and airway tissues of mice in Control, *B. pertussis*, sh-NC + Control, sh-MASP1 + Control, sh-NC + *B. pertussis*, and sh-MASP1 + *B. pertussis* groups at different time points; (**F**) Representative images of lung tissue stained with H&E and pathological score statistics at 7 days post-infection (scale bar = 25 µm); (**G-H**) Expression of relevant factors in lung and airway tissues of mice in each group at 7 days post-infection detected by RT-qPCR and Western blot; each group *n* = 10; ^ns^*P* >0.05, *P < 0.05, **P < 0.01, ***P < 0.001.

Further, we conducted *in vivo* experiments to validate the role of MASP1 in *B. pertussis* infection. As described in the Methods section, we established a *B. pertussis* infection model in mice and assessed MASP1 expression in lung and airway tissues at various time points. The results indicated that MASP1 expression was significantly upregulated in the lung and airway tissues on days 4, 7, and 14 post-infection (Fig. 5A through C).

Additionally, we investigated the effect of MASP1 inhibition on the *B. pertussis* infection group. Lentiviral vectors were used to suppress MASP1 expression in lung and airway tissues (Fig. S8A and B), and relevant indicators were measured post-infection. Results showed that compared with the sh-NC + Control group, the bacterial load in the lung and airway tissues of the sh-NC + *B. pertussis* group significantly decreased on days 4, 7, and 14. The bacterial load was highest on day 7 in the sh-NC + *B. pertussis* group but significantly reduced in the sh-MASP1 + *B. pertussis* group (Fig. 5D and E). Therefore, on day 7, we further examined relevant indicators. Lung tissue analysis showed that the sh-MASP1 + *B. pertussis* group had significantly less lung tissue damage compared with the sh-NC + *B. pertussis* group (Fig. 5F). Analysis of inflammatory cytokines and antimicrobial peptides revealed that in lung and airway tissues, TNF-α and IL-1β expression was downregulated, and IL-10 and BD-2 expression was significantly upregulated in the sh-MASP1 + *B. pertussis* group compared with the sh-NC + *B. pertussis* group (Fig. 5G and H), indicating that MASP1 inhibition could alleviate symptoms of *B. pertussis* infection in mice.

In summary, the results demonstrate that MASP1 expression is upregulated in the lung and airway tissues of *B. pertussis*-infected mice, and that MASP1 inhibition can alleviate the symptoms caused by *B. pertussis* infection.

## DISCUSSION

This study utilized multi-omics analysis to reveal significant differences in nasal and pharyngeal microbial diversity and specific microbial abundances between pediatric patients with severe pertussis and healthy children. These findings align with previous research on pertussis infection’s impact on the nasal and pharyngeal microbiota, but this study goes further by providing more detailed information on microbial composition and functionality through metagenomic and 16S rRNA data (34, 35). These discoveries not only offer a new perspective on pertussis-related microbial ecological changes but also lay a theoretical foundation for developing intervention strategies based on microbiota modulation. For instance, while previous studies mostly focused on changes in bacterial communities, this study encompasses a broader microbial spectrum, including viruses and fungi, thus enriching the understanding of the dynamic fluctuations in the nasal and pharyngeal microbiota.

This study, for the first time, employed a combination of multi-omics and machine learning methods to screen and validate MASP1 as a characteristic gene with expression changes in pertussis. While past research primarily focused on the role of MASP1 in other infectious diseases, this study uncovered, for the first time in the context of pertussis, the crucial regulatory role of MASP1. Particularly, the significant upregulation of MASP1 in severe pertussis patients suggests its potentially important role in disease progression, offering a foundation for further exploration of MASP1’s universal mechanisms in infectious diseases. In contrast to the emphasis in other studies on overall changes in host immune responses, this study, through detailed analysis, elucidated the specific cellular and molecular impacts of MASP1.

This study investigated the role of MASP1 in pertussis infection using the human bronchial epithelial cell line HBE135-E6E7 *in vitro*. By manipulating MASP1 expression levels through overexpression and knockdown, the study confirmed MASP1’s function in pertussis infection. The results demonstrated that MASP1 overexpression exacerbated pertussis-induced cell damage, adding to the understanding of MASP1’s involvement in cellular inflammatory responses from previous studies. While MASP1 has traditionally been recognized as an immune response regulator, this research further established its direct role in airway epithelial cell injury (36–38). This deepened insight into MASP1’s cellular actions not only paves the way for potential cell protection strategies but also sheds light on our comprehension of its function at the cellular level.

Using a mouse model of pertussis infection, this study evaluated the impact of inhibiting MASP1 expression on infection symptoms. The findings revealed that MASP1 inhibition significantly alleviated symptoms caused by pertussis bacteria, reducing bacterial burden and tissue damage in the lungs and airways. This aligns with prior research on MASP1’s role in inflammatory responses and, for the first time in an animal model, validates the potential of MASP1 as a therapeutic target (39, 40). These discoveries suggest that through targeted MASP1 regulation, the severity of pertussis infection can be effectively controlled, offering new avenues for future clinical treatments.

This study reveals that children with pertussis exhibit significantly decreased nasal and pharyngeal microbial diversity compared with healthy children, with an increase in the abundance of specific pathogenic bacterial groups. This finding aligns with previous research on the impact of infectious diseases on microbial diversity. However, utilizing more refined metagenomic data, this study further elucidates specific changes in bacterial communities and their association with disease severity (7, 41). For instance, an increase in the abundance of specific bacterial groups, such as *B. pertussis*, correlates positively with disease severity (1, 2). Clinical treatment guidelines indicate that macrolide antibiotics (such as erythromycin, clarithromycin, or azithromycin) have consistently been effective and are among the primary treatment options for pertussis patients. Short-term antibiotic treatments (azithromycin for 3 to 5 days or clarithromycin or erythromycin for 7 days) are as effective as long-term erythromycin treatment (10 to 14 days) in eradicating *B. pertussis* from the nasopharynx. Therefore, routine antibiotic treatment may be one of the key factors contributing to the greater diversity of nasopharyngeal microbiota in children with pertussis (42). Consequently, the aforementioned bioinformatic investigation offers new insights for future microbiota-targeted intervention strategies, suggesting that modulating microbiota composition could provide an avenue for early-stage intervention in the progression of pertussis.

Furthermore, through the application of machine learning algorithms, this study successfully identified the key gene MASP1 associated with pertussis. This approach represents a novel application of machine learning in infectious disease research, showcasing the significant potential of machine learning in big data analysis and biomarker discovery. While prior studies heavily relied on traditional bioinformatics analyses, this study’s integration of machine learning enhances the precision and efficiency of gene identification (37). This method is not only applicable to pertussis research but also provides a reference for identifying key genes in other infectious diseases.

**Fig 6:**
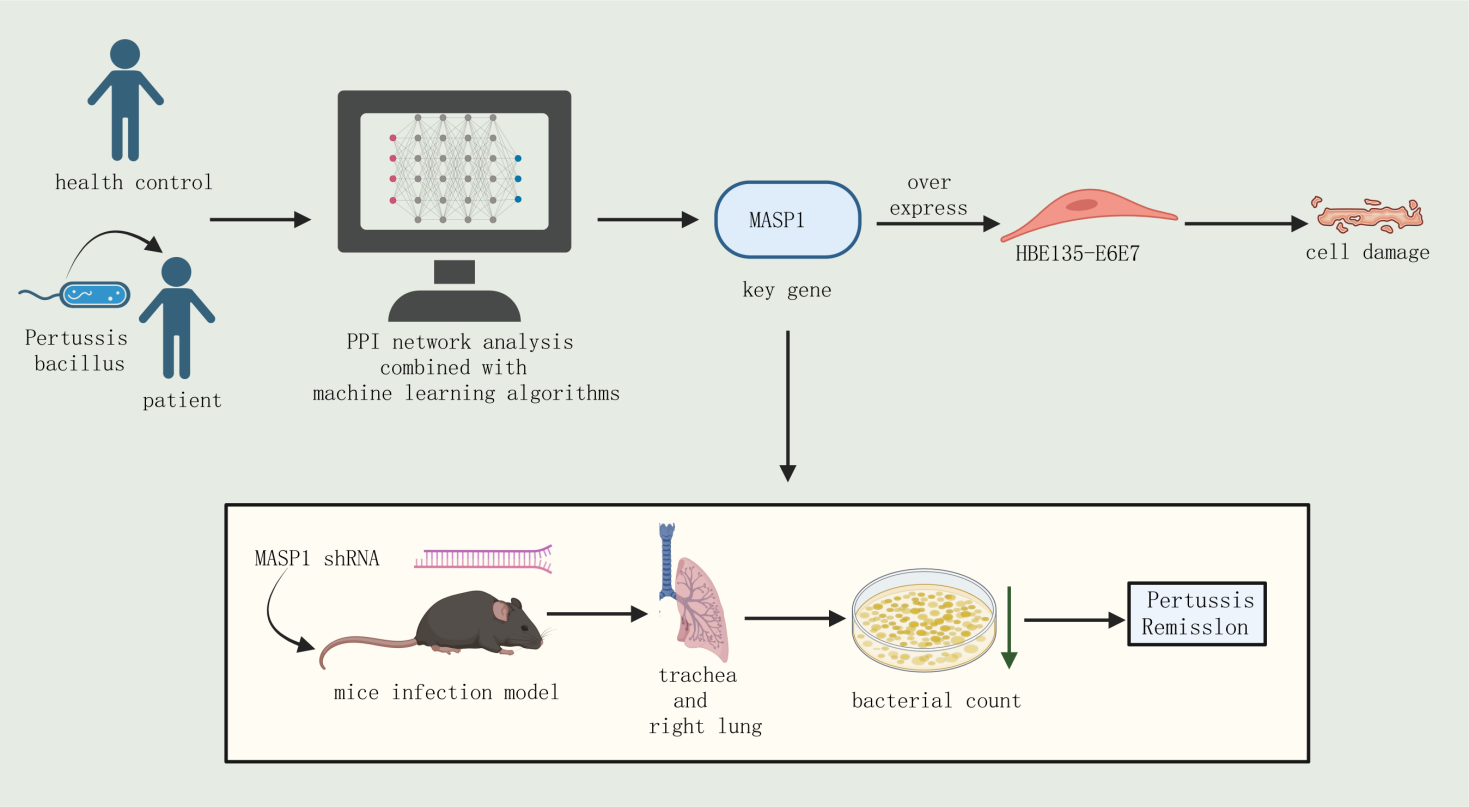
Inhibition of MASP1 alleviates epithelial cell damage induced by *B. pertussis* and symptoms of infection in mice.

This study utilized multi-omics analysis to uncover the crucial role of MASP1 in severe pertussis in children (Fig. 6). The analysis revealed significant differences in nasal and pharyngeal microbial diversity and abundance of specific bacterial groups between healthy children in the control group and pediatric patients with pertussis. Further investigation indicated an upregulation of MASP1 expression in airway epithelial cells post-infection. *In vitro* cell experiments demonstrated that MASP1 expression increased in HBE135-E6E7 cells following pertussis infection, and overexpression of MASP1 exacerbated *B. pertussis*-induced damage to HBE135-E6E7 cells. Both *in vitro* and *in vivo* experimental outcomes suggested that inhibition of MASP1 could alleviate the symptoms caused by *B. pertussis* infection. These results underscore the critical role of MASP1 in pertussis infection.

By integrating multi-omics analysis and experimental validation, this study elucidated the pivotal role of MASP1 in severe pertussis among children, substantiating the potential of inhibiting MASP1 expression to significantly alleviate pertussis symptoms. This discovery not only enhances the understanding of pertussis pathogenesis but also provides a novel perspective for the academic community and lays a solid theoretical foundation for the development of therapeutic strategies targeting MASP1 in clinical settings. Specifically, interventions targeting MASP1 present a promising avenue for treating severe pertussis, potentially lowering the complications and mortality associated with infections, thereby enhancing patients’ quality of life. Furthermore, the successful application of multi-omics and machine learning methods in this study provides valuable experience and technical support for the identification of key genes and targeted therapies in infectious disease research, offering insights for potential applications in the screening and treatment of other diseases.

While this study has made significant discoveries, there are limitations that need to be addressed in future research. First, the relatively small sample size in this study warrants further validation of the research findings in a larger population. Second, although both *in vitro* and *in vivo* experiments suggest that inhibiting MASP1 can help alleviate pertussis symptoms, more clinical trials are needed to confirm the applicability of these results to humans. Furthermore, as this study primarily focused on children with severe pertussis, excluding other age groups or patients with milder symptoms may limit the generalizability of the results. Finally, while this study has uncovered the critical role of MASP1 in pertussis, further research is required to delve into its specific molecular mechanisms and regulatory pathways.

Future research should aim to expand the sample size, encompassing pertussis patients of different age groups and varying severity of the disease, to validate the universality and efficacy of targeting MASP1 as a treatment approach. Moreover, advancing clinical trials to evaluate the safety and effectiveness of treatment strategies based on MASP1 inhibition in clinical practice is essential. Additionally, in-depth exploration of the specific molecular mechanisms and signaling pathways of MASP1 in the pathogenesis of pertussis can lead to the discovery of more potential therapeutic targets, refining existing treatment approaches. By integrating multi-omics data and advanced machine learning algorithms, similar methods can be applied in screening key genes in other infectious diseases, promoting the development of personalized precision medicine. Ultimately, the outcomes of this study hold promise for improving the treatment outcomes of pertussis, reducing its public health burden, and providing new scientific evidence and clinical guidance.

## MATERIALS AND METHODS

### Acquisition of 16S rRNA sequencing data

Phenotypic information for all samples related to childhood pertussis projects was retrieved from the EMBL-EBI database (https://www.ebi.ac.uk/ena/browser/search) under the biological project number PRJEB45720. Subsequently, sequencing data for this project, comprising nasal–pharyngeal samples from nine healthy children in the control group and 12 children diagnosed with pertussis, were downloaded from the NCBI Sequence Read Archive (SRA) database (https://www.ncbi.nlm.nih.gov/sra/).

### Analysis of nasopharyngeal microbial diversity and species composition

Initially, open-source software VSEARCH (vsearch-2.8.1-win-x86_64) and Usearch (usearch10.0.240_win32) were utilized within R for processing the raw reads data, encompassing sequence merging, denoising, clustering, and feature table construction, to facilitate subsequent OTU/ASV analyses. Following this pre-processing, the “vegan” package was employed to perform rarefaction for evaluating species complexity diversity in the samples. Alpha diversity analysis was conducted using richness, Chao1, ACE, Shannon, Simpson, and inverse Simpson indices. A distance matrix heatmap based on Bray–Curtis dissimilarity was generated to visually illustrate the similarity/dissimilarity among samples, aiding in classification clustering and trend analysis. Principal coordinates analysis (PCoA) was applied to the beta diversity assessment. Wilcoxon rank sum tests and Welch *t*-tests were performed to compare bacterial abundance and diversity. Differential abundance analysis between groups was calculated using the R package edgeR, followed by volcano plots and visualization of Manhattan plots. The LDA effect size (LEfSe) analysis was employed to generate bar charts displaying all differential abundances, with a linear discriminant analysis (LDA) score threshold set at 2.0. LDA scores indicate the impact of significantly different species between groups, with higher scores reflecting greater dissimilarities in characteristics (43).

### Analysis of functional composition of nasopharyngeal flora

The tub data were analyzed using the online platform ImageGP to predict the macro-genomic pathways of each primer set based on the Kyoto Encyclopedia of Genes and Genomes (KEGG) database (44, 45). Subsequently, STAMP software (v2.1.3) was utilized for statistical analysis and visualization of the non-hierarchical results. *t*-tests were conducted to compare differences in functional composition.

### Transcriptome data acquisition and preprocessing

The pertussis (Cryptorchidism)-related transcriptome sequencing data GSE182807 was obtained from the Gene Expression Omnibus (GEO, https://www.ncbi.nlm.nih.gov/geo/). The data set GSE182807 consisted of three samples of normal human airway epithelial cells and nine samples of human airway epithelial cells stimulated with *B. pertussis*. Differential gene expression analysis was performed using the “limma” package in R language to identify the differentially expressed genes (DEGs). The DEGs between the normal human airway epithelial cell samples and *B. pertussis*-stimulated human airway epithelial cell samples in the GSE182807 data set were selected based on the criteria of |logFC| > 1 and *P* < 0.05. The R package pheatmap was utilized to generate expression heatmaps and volcano plots of the DEGs (46).

### Protein–protein interaction analysis (PPI)

The selected potential therapeutic targets were input into the String database platform (https://string-db.org/) for PPI analysis, with *Homo sapiens* chosen as the target species. The confidence score for interactions was set at 0.170, unconnected nodes were hidden, and other parameters were kept at default settings to generate a PPI network for potential pertussis treatment targets. The protein interaction data were downloaded for further analysis. Subsequently, the R software was used to create a bar graph depicting the node connectivity of each protein in the network (47).

### Machine learning for key gene set selection

Three machine learning methods, namely least absolute shrinkage and selection operator (LASSO), support vector machine (SVM), and random forest (RF), were utilized to select a set of key genes associated with the disease. LASSO regression analysis was conducted using the glmnet package (version 4.0–2) in the R language, SVM analysis was performed with the e1071 package (version 1.7–3), and RF analysis was carried out using the randomForest package (version 4.6–14). The data set was divided into training and testing sets in a 70% to 30% ratio for model evaluation (48, 49).

### Cell culture and lentivirus infection

Human bronchial epithelial cell line HBE135-E6E7 (CRL-2741, ATCC, USA) was cultured in HBE135-E6E7 specific cell culture medium (CM-0732, Pricella, Wuhan, China) at 37°C in a humidified atmosphere with 5% CO_2_.

The overexpression and knockdown cell lines of MASP1 were constructed in HBE135-E6E7 cells using lentivirus-mediated methods, along with corresponding control groups (oe-NC and oe-MASP1, sh-NC, and sh-MASP1). Plasmids and lentivirus packaging services were provided by GeneTech Bioengineering (Shanghai, China). In brief, plasmids carrying the target gene sequence/shRNA sequence were co-transfected into 293T cells (CRL-3216, ATCC, USA) along with helper plasmids. After validation, amplification, and purification, packaged lentivirus was obtained. For lentivirus-mediated cell transfection, cells were seeded at a density of 5 × 10^5^ cells/well in a six-well plate. When the cell confluency reached 50%–70%, transfection was performed using DMEM/F-12 medium (11320033, Thermo Fisher, USA) containing an appropriate amount of packaged lentivirus (MOI = 10, working titer about 5 × 10^6^ TU/mL) and polybrene (5 µg/mL). After 4 h of transfection, an equal amount of medium was added to dilute polybrene, followed by medium replacement after 24 h and observation of transfection efficiency through fluorescence reporter gene analysis after 48 h. Subsequently, puromycin (1 µg/mL) was used for resistance selection to obtain stably transfected cell lines (50). Knockdown cell line construction employed two different shRNA sequences, with the more efficient sequence chosen for subsequent experiments, Table S1 for detailed shRNA sequences.

### Cultivation of *B. pertussis* and infection of HBE135-E6E7 cells

The *B. pertussis* strain (9797, ATCC, USA) was cultured on Bordet–Gengou agar plates (Becton Dickinson) containing 15% defibrinated sheep blood. Colonies from fresh plates were suspended in a modified Stainer–Scholte medium (supplemented with 1 g/L casamino acids and 1 g/L 2-hydroxypropyl-β-cyclodextrin) to achieve an optical density of 0.2 at 600 nm (OD 600). The bacteria were then allowed to grow overnight with agitation at 37°C until reaching an OD 600 of 1 (2 × 10^9^ CFU/mL). The bacterial suspension was diluted to 2 × 10^7^ CFU/mL in DMEM/F-12 medium containing 10% fetal bovine serum (10100147C, Thermo Fisher, USA) without antibiotics and further incubated at 37°C for 1 h before being added to HBE135-E6E7 cells at a multiplicity of infection (MOI) of 50. Uninfected HBE135-E6E7 cells were used as the control group (Control), while *B. pertussis*-infected HBE135-E6E7 cells were designated as the infected group (*B. pertussis*) (33), in line with the design of the manuscript.

### Cultivation at the air-liquid interface and detection of transepithelial electrical resistance (TEER)

In the air–liquid interface (ALI) cultivation, human bronchial epithelial cell lines were able to form a pseudostratified epithelium, establish functional tight junctions, and respond to bacterial components.

The ALI culture was established on Transwell permeable filter supports. Cells were seeded in a cell-specific culture medium for HBE135-E6E7 containing antibiotics. After 3–4 days post-seeding, the medium was replaced with DMEM-F/12 supplemented with 2% Ultroser G and antibiotic solution on the apical and basolateral sides. After another 3–4 days, the medium was removed from the apical surface, and the cells were maintained at the ALI for 21 days with daily medium changes. Matured cultures of HBE135-E6E7 cells at the ALI exhibited a TEER of at least 350 Ω × cm², which was crucial for further investigations.

TEER measurements were conducted using a Millicell-ERS-2 voltohmmeter (MERS00002, Merck, Germany). For the *B. pertussis* infection experiments, bacteria were resuspended in DMEM/F-12 containing 10% FBS without antibiotics, and added to the apical surface of HBE135-E6E7 cell monolayers. The background resistance of the empty Transwell permeable inserts was subtracted. TEER was calculated and expressed in Ω × cm² (33).

### Immunofluorescence staining

Cells on the Transwell membrane were washed twice with ice-cold PBS. Fixation was performed using cold methanol (−20°C) for 15 min, followed by permeabilization with acetone (−20°C) for 50 s, and an additional wash with methanol. The cells were then rehydrated at 4°C for 5 min in methanol solutions of 70%, 50%, and 30%, sequentially. After fixation, the cell layer was washed with PBS and blocked with 5% bovine serum albumin (BSA) in PBS at room temperature for 30 min. Subsequently, the membrane containing the cell layer was carefully detached from the polystyrene support using sharp forceps. Primary antibodies, anti-ZO1 Ab (rabbit anti-mouse, 1:100) (ab276131, Abcam, UK), and anti-*B. pertussis* Ab (mouse anti-*B. pertussis*, 1:80) (MA1-7005, Thermo Fisher, USA), were diluted in 2% BSA-PBS and incubated at room temperature for 60 min, followed by three washes with PBS.

After washing, fluorescent secondary antibodies Goat Anti-Rabbit IgG H&L (Alexa Fluor 488) (1:1000) (ab150077, Abcam, UK) and Goat anti-Mouse IgG3 cross-adsorbed secondary antibody, Alexa Fluor 594 (10 µg/mL) (A-21155, Thermo Fisher, USA) were applied and incubated at room temperature for 1.5 h. The cells were then washed three times with PBS and counterstained with DAPI (62248, Thermo Fisher, USA) for 10 min. Finally, an anti-fade mounting medium was used to mount the membrane (51). The staining results were observed and recorded under a laser scanning confocal fluorescence microscope (STELLARIS 5, Leica, Germany).

The tight junction organization rate (TiJOR) was calculated using Image-Pro Plus 6.0 (Media Cybernetics, USA) to evaluate the integrity of the tight junction network. TiJOR was determined by analyzing the entire representative image obtained from confocal microscopy. Untreated cell layer regions were randomly selected as controls to optimally present the tight junction networks (52).

### ELISA detection of cytokines and cAMP

Cell culture medium supernatants were collected and analyzed using enzyme-linked immunosorbent assay (ELISA) kits according to the manufacturer’s instructions to measure the expression levels of IL-10 (ab185986, Abcam, UK), TNF-α (ab181421, Abcam, UK), IL-1β (ab214025, Abcam, UK), and BD-2 (900-T172, Thermo Fisher, USA) in the samples (53).

Cells were lysed using a 50 mM HCl solution containing 0.2% Tween, followed by the assessment of cAMP levels using a cAMP ELISA kit (ab290713, Abcam, UK). The cAMP concentrations were normalized to total protein content, and protein concentrations were measured using a BCA assay kit (P0012S, Bestbio, Shanghai, China) (33).

### Construction and management of *B. pertussis* infection model in mice

Two hundred and seventy female C57BL/6 J mice, aged 6–8 weeks and weighing 20 ± 2 grams, were purchased from Beijing Vital River Laboratory Animal Technology Co., Ltd. (located in Beijing, China). The mice were housed in standard cages under controlled environmental conditions, at a temperature of 23 ± 1°C and a 12 h light–dark cycle. Food and water were provided *ad libitum*. Prior to the experiments, the mice underwent a 1-week acclimatization period. The experimental procedures and animal usage protocols were approved by our institution’s Animal Ethics Committee. All animal experiments in this study adhered to internationally recognized animal welfare standards and relevant regulations. Every possible measure was taken during the experiments to minimize animal discomfort and distress. At the conclusion of the experiments, the animals were humanely euthanized.

During *B. pertussis* infection, mice were anesthetized with inhaled isoflurane and intranasally inoculated with 50 µL of *B. pertussis* suspension containing approximately 1 × 10^6^ CFU of bacteria (54). For lentivirus treatment, MASP1 shRNA (CCTGTCCCTATGACTACATTA) lentiviral vector (1 × 10^8^ infectious units [IFU]) was administered via tracheal intubation to anesthetized animals 2 days prior to bacterial inoculation, with sh-NC as a control (55). At specified time points, mice were euthanized by inhalation of carbon dioxide followed by thoracotomy to harvest relevant tissues for subsequent experiments.

Mice in the MASP1 expression detection experiment were divided into uninfected (Control) and infected (*B. pertussis*) groups based on bacterial load in lung and airway tissues. Lung and airway tissue samples were collected on days 1, 4, 7, and 14 post-infection for analysis, with a sample size of *n* = 10 per group per time point. For the MASP1 lentivirus knockdown experiment, the groups included the PBS-treated group (Mock), sh-NC, and sh-MASP1 treated groups. Lung and airway tissue samples were harvested 2 days after lentivirus treatment to assess MASP1 expression, with *n* = 10 in each group. To validate the impact of MASP1 knockdown on the severity of *B. pertussis* infection, the experimental groups included Control, sh-NC + Control, sh-MASP1 + Control, *B. pertussis*, sh-NC + *B. pertussis*, and sh-MASP1 + *B. pertussis*. Lung and airway tissue samples were collected on days 1, 4, 7, and 14 post-infection for bacterial load detection. Additionally, tissue samples collected on day 7 post-infection were analyzed for relevant factors. The sample size for each group was *n* = 10.

For bacterial counting analysis, the trachea and right lung lobes (cranial, medial, caudal, and accessory lobes) were immersed in 2 mL of sterile PBS and homogenized. Serial dilutions of the homogenates were spread on BG blood agar plates containing 200 μg/mL of chloramphenicol. Bacterial colonies were counted after incubation at 37°C for 4 to 5 days to determine colony-forming units (CFU) (56).

### H&E staining and pathological scoring

Immunohistochemical analysis was conducted on samples from the left lung. Following the harvesting of lung tissue for histological analysis, heart perfusion was carried out using PBS, and the right lung (including the cranial lobe, medial lobe, caudal lobe, and accessory lobe) was ligated and removed for further analysis. The left lung was infused through the tracheal incision with 10% (w/v) buffered formalin (Sigma), then stored in 10% (w/v) buffered formalin and sectioned after embedding.

For H&E staining of the tissues, the sections were first rinsed in 1 × PBS for 2 s, followed by hematoxylin staining at 60°C for 60 s. Subsequently, they were rinsed in 1 × PBS for 10 s, then treated with 1% hydrochloric acid alcohol differentiation solution for 3 s, followed by another 2 s rinse in 1 × PBS. Next, the sections were stained with eosin for 3 min, rinsed again in 1 × PBS for 2 s, dehydrated in 70%, 80%, 95% ethanol, and absolute ethanol for 5 min each, followed by three treatments with xylene for 5 min each, and finally mounted with resin for slide sealing. The staining results were observed and recorded using an optical microscope (Olympus, CX43, manufactured in Japan).

The tissue pathological scoring ranged from 0 to 3, with a grade of 3 indicating the most severe pathological presentation. The scoring criteria included the following items: (i) degree of inflammation in broncho-vascular bundles (BVB), (ii) percentage involvement of BVB, and (iii) degree of pleural inflammation. The total score could reach up to nine points. A higher score indicated a more severe degree of pathological injury (56).

### RT-qPCR

Total RNA from samples was extracted using Trizol, followed by one-step reverse transcription and PCR using the One Step TB Green PrimeScript RT-PCR Kit (RR066A, Takara, Japan). The RT-qPCR reactions were carried out in the Thermal Cycler Dice Real-Time System III (TP990, Takara, Japan). The thermal cycling program included a reverse transcription stage (42°C for 5 min, 95°C for 10 s, one cycle), a PCR stage (95°C for 5 s, 60°C for 34 s, 40 cycles), and a melting curve stage (95°C for 15 s, 60°C for 1 min, 95°C for 15 s, one cycle), followed by confirmation of the amplification and melting curves. The fold change in the expression of the target gene between the experimental and control groups was calculated using the 2^-ΔΔCt^ method, where ΔΔCT = ΔCt _test_ - ΔCt _control_, and ΔCt = Ct _target_ - Ct _reference_. Ct represents the cycle threshold at which the real-time fluorescence signal reaches a set threshold (51). Each sample was run with triplicate wells, and the experiment was repeated three times. Primer sequences are listed in Table S2, with β-actin serving as the internal reference gene.

### Western blot

Total protein from samples was extracted using a protein extraction kit (BB3101, Bestbio, Shanghai, China), and the protein concentration was determined with a BCA assay kit (P0012S, Bestbio, Shanghai, China). A 10% SDS-PAGE gel (P0012A, Bestbio, Shanghai, China) was prepared for the experiment. Each well was loaded with 50 µg of protein samples, followed by electrophoresis at a constant voltage of 80 V for 2 h, increasing to 120 V at 250 mA constant current for 90 min to transfer the proteins to a PVDF membrane (IPVH00010, Merck, Germany). The PVDF membrane was then blocked with 5% skim milk in TBST at room temperature for 2 h, washed with TBST for 10 min to remove excess reagents, and then incubated overnight at 4°C with the appropriate primary antibody. After washing with TBST to remove excess primary antibody, the membrane was incubated at room temperature for 1 h and then incubated with Goat Anti-Rabbit IgG H&L (HRP) (1:2000) (ab6721, Abcam, UK) or Goat Anti-Mouse IgG H&L (HRP) (1:2000) (ab6789, Abcam, UK), followed by another wash to remove excess secondary antibody. Finally, the membrane was developed using an ECL reaction kit (P0018FS, Bestbio, Shanghai, China), exposed in a dark box, and visualized. β-actin was used as an internal control, and the results were quantitatively analyzed using Image-Pro Plus 6.0 (Media Cybernetics, USA) (57). Each experiment was replicated three times per sample. For details regarding the antibodies used, please refer to Table S3.

### Statistical analysis

The data, derived from at least three independent experiments, are presented as mean ± standard deviation (mean ± SD); for comparisons between the two groups, a two-sample independent *t*-test was employed; for comparisons involving three or more groups, one-way analysis of variance (ANOVA) was used. If the ANOVA results indicated significant differences, a Tukey’s honestly significant difference (HSD) post-hoc test was conducted to compare differences between groups. For non-normally distributed or unequal variance data, the Mann–Whitney U test or Kruskal–Wallis H test is applied. All statistical analyses were performed using GraphPad Prism 9 (GraphPad Software, Inc.) and R language. The significance level for all tests was set at 0.05, with a two-tailed *P*-value less than 0.05 considered statistically significant.

## Data Availability

All data can be provided upon request.

## SUPPLEMENTAL MATERIAL

Supplemental material iai.00271-24-s0001.pdf Tables S1 to S3; Fig. S1 to S8.

ASM does not own the copyrights to Supplemental Material that may be linked to, or accessed through, an article. The authors have granted ASM a non-exclusive, world-wide license to publish the Supplemental Material files. Please contact the corresponding author directly for reuse.
