# Supplementary material for: MASP1 modulation as a novel therapeutic target in severe pediatric pertussis: insights from a multi-omics approach
Source: Infect Immun. 2025 Jan 22;93(2):e00271-24. doi: 10.1128/iai.00271-24 (PMC11834402; doi:10.1128/iai.00271-24)
Supplement: Supplemental material — Tables S1 to S3; Fig. S1 to S8. [file iai.00271-24-s0001.pdf]

Table S1. shRNA sequences.

Name	sh-RNA Sequences (5'-3')
sh-NC	CCTAAGGTTAAGTCGCCCTCG
sh-MASP1-1 (human)	GCCCTATTACAAGATGCTCAA
sh-MASP1-2 (human)	CCAGTGATTCAGAGGTGACTT

Table S2. RT-qPCR primer sequences.

Genes (species)	Sequences (5'-3')
MASP1 (human)	F: CACCGTGGAGCTAAACAATATGT
	R: GCTTGATCCGAAACCCATCTG
MASP1 (mouse)	F: ACCGTGGAGCTAAACGAAATG
	R: TCCAAGTTGAAGTGCATGAAGT
IL-10 (human)	F: TCAAGGCGCATGTGAACTCC
	R: GATGTCAAACTCACTCATGGCT
IL-10 (mouse)	F: CTTACTGACTGGCATGAGGATCA
	R: GCAGCTCTAGGAGCATGTGG
TNF-α (human)	F: CCTCTCTCTAATCAGCCCTCTG
	R: GAGGACCTGGGAGTAGATGAG
TNF-α (mouse)	F: CAGGCGGTGCCTATGTCTC
	R: CGATCACCCCGAAGTTCAGTAG
IL-1β (human)	F: ATGATGGCTTATTACAGTGGCAA
	R: GTCGGAGATTCGTAGCTGGA
IL-1β (mouse)	F: GAAATGCCACCTTTTGACAGTG
	R: TGGATGCTCTCATCAGGACAG
BD-2 (human)	F: CTCCTCTTCTCGTTCCTCTTCA
	R: GCAGGTAACAGGATCGCCTAT
BD-2 (mouse)	F: TGGAGTCTGAGTGCCCTTTC
	R: AGTGGTCAAGTTCTGCTTCGT
β-actin (human)	F: GAGAAAATCTGGCACCACACC
	R: GGATAGCACAGCCTGGATAGCAA
β-actin (mouse)	F: GTGACGTTGACATCCGTAAAGA

R: GCCGGACTCATCGTACTCC

Note: F: forward; R: reverse.

Table S3. Western blot antibody information.

Targets (host and reactivity)	Manufacturer	Cat.No.	Tested Dilution
MASP1 (rabbit anti-human/mouse)	Abcam	ab232945	2 μg/mL
ZO1 (rabbit anti-human)	Abcam	ab276131	1:1000
Occludin (rabbit anti-human)	Abcam	ab216327	1:1000
Claudin 1 (rabbit anti-human)	Abcam	ab307692	1:1000
IL-10 (rabbit anti-mouse)	Abcam	ab310329	1:1000
TNF-α (rabbit anti-mouse)	Abcam	ab183218	1:1000
IL-1β (rabbit anti-mouse)	Abcam	ab283818	1:1000
BD-2 (rabbit anti-mouse)	Thermo Fisher	PA5-75665	1:500
β-actin (mouse anti-mouse/human)	Abcam	ab6276	1:5000

Note: Abcam, UK; Thermo Fisher, USA.

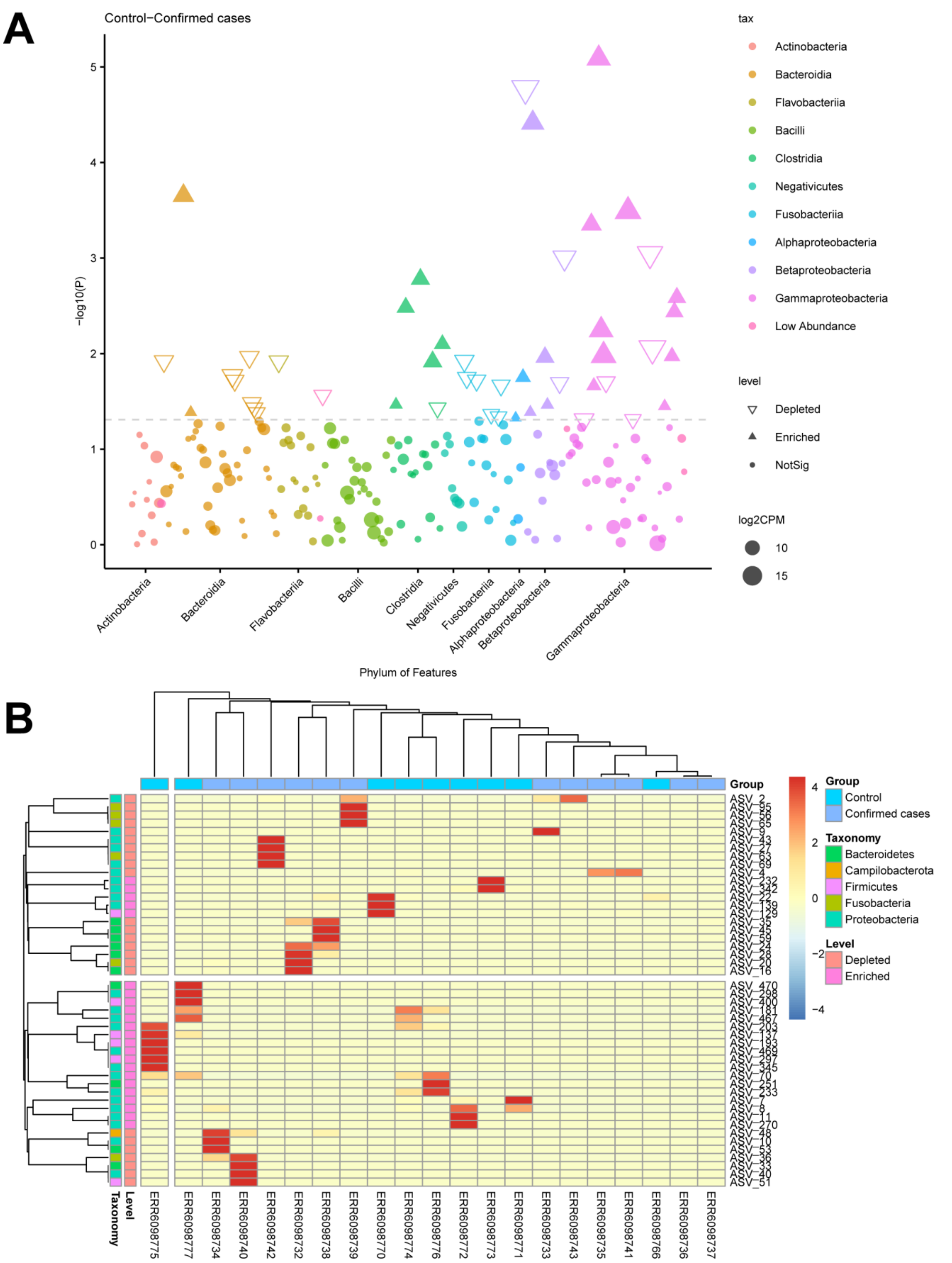

Figure S1. Comparison of nasopharyngeal flora abundance between control and Confirmed cases group.

Note: (A) Manhattan plot comparing inter-group abundance differences between Control and Confirmed cases group; (B) Heat map of inter-group abundance differences between Control and Confirmed cases group. Control group (n = 9), Confirmed cases group (n = 12).

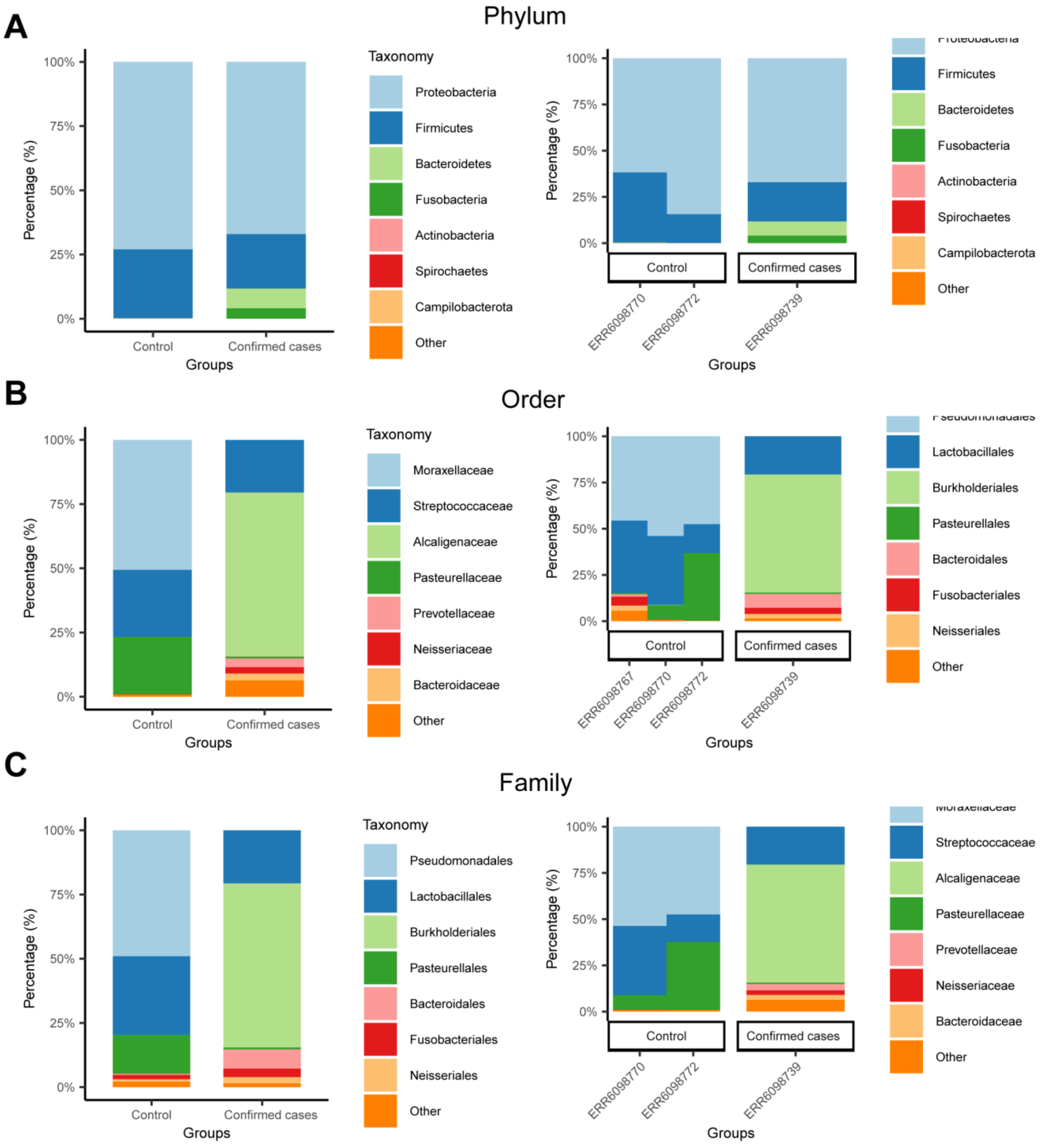

Figure S2. Comparison of nasopharyngeal flora abundance differences between control and Confirmed cases group.

Note: (A-C) Stacked bar plots of nasopharyngeal flora species abundance in Control (n=9) and Confirmed cases (n=12) groups at Phylum, Order, and Family levels, where the left shows stacked bar graphs of mean species abundance, and the right displays species abundance stacked bar graphs combined with sample grouping.

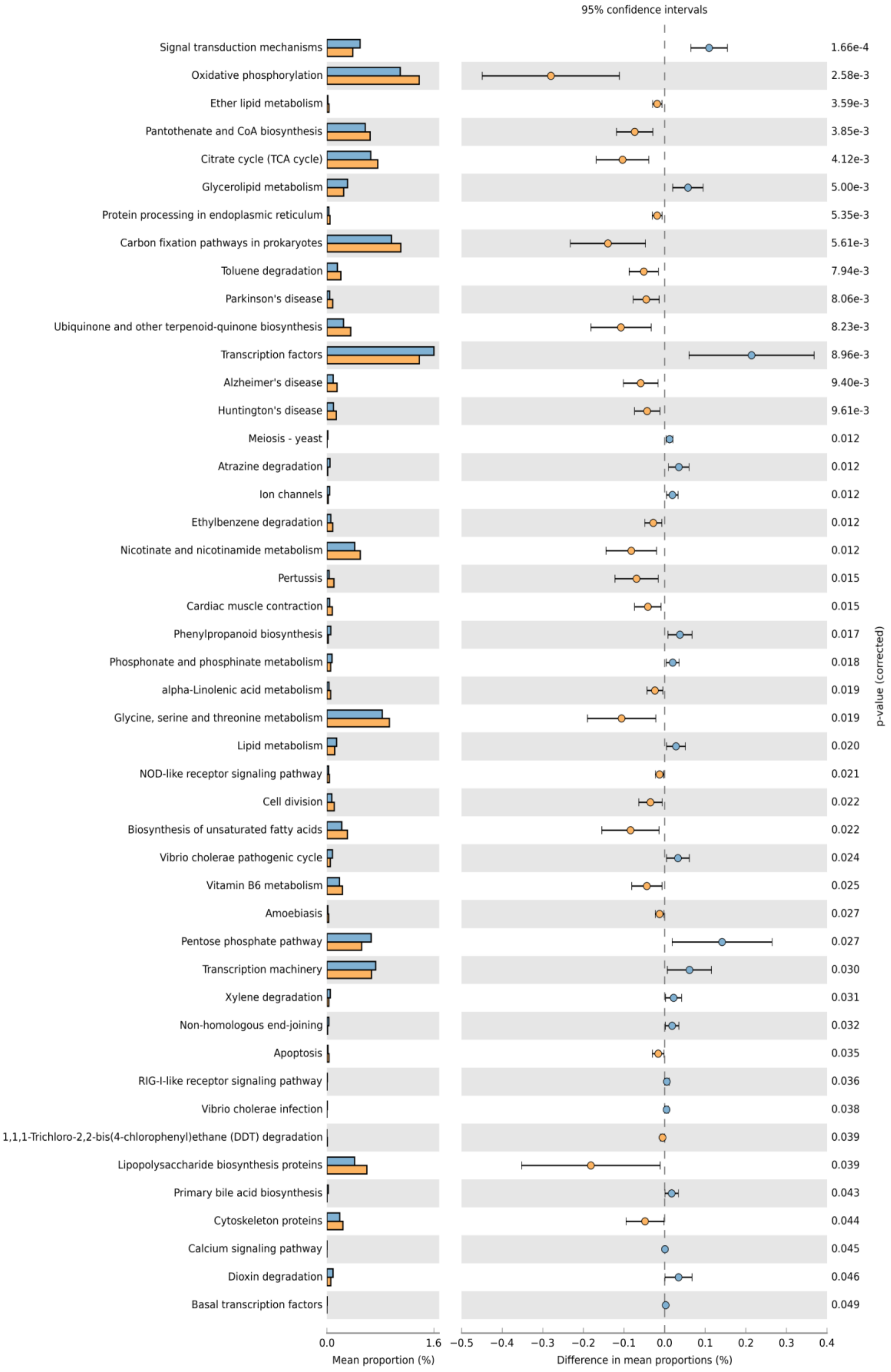

Figure S3. Functional enrichment analysis of nasopharyngeal flora in control and Confirmed cases group.

Note: Functional enrichment analysis of nasopharyngeal flora in Control (n=9) and Confirmed cases (n=12) groups ($P < 0.05$).

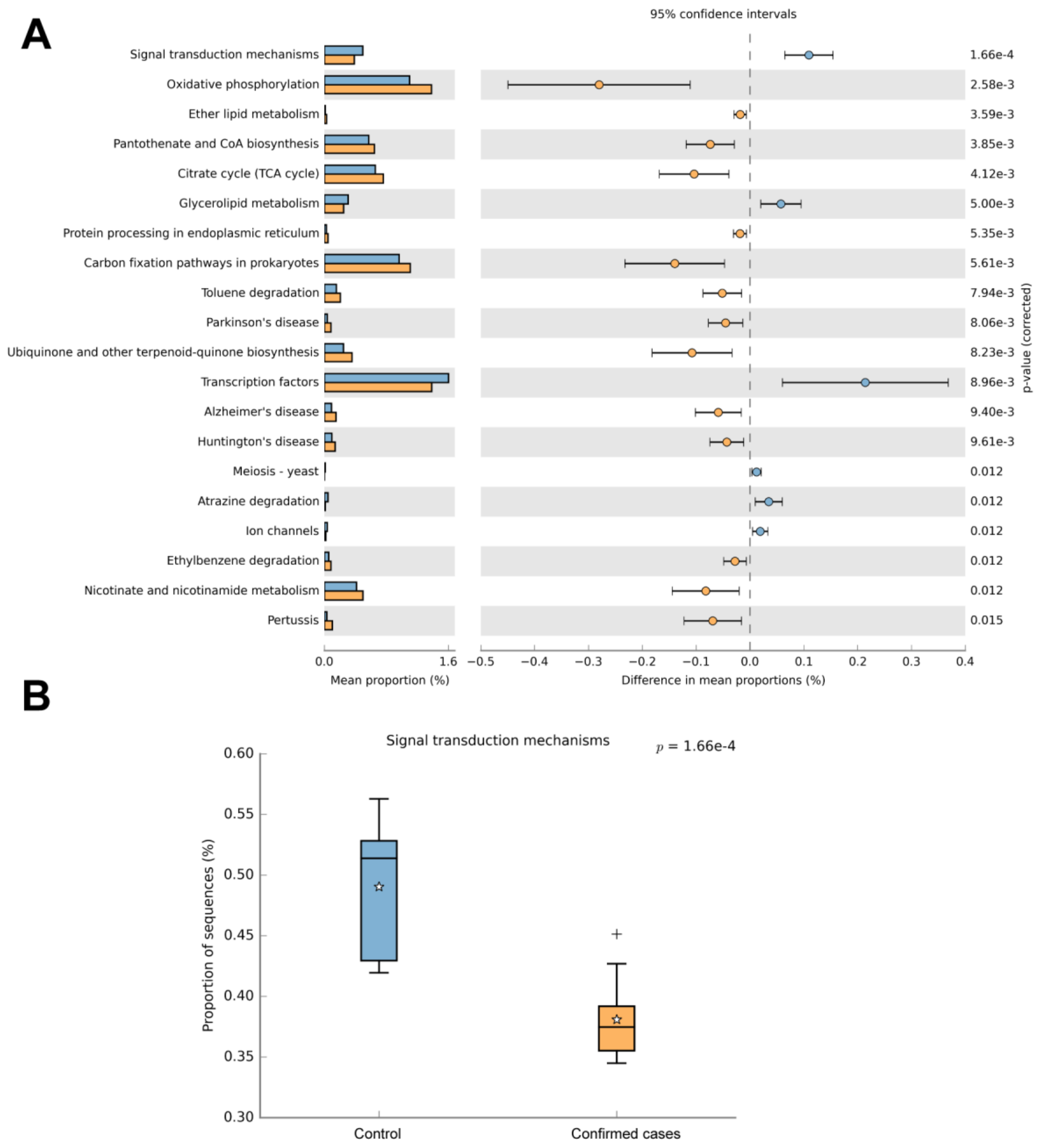

Figure S4. Functional enrichment analysis of Top 20 nasopharyngeal flora in control and Confirmed cases group.

Note: (A) Functional enrichment analysis (*P* value, TOP 20) of nasopharyngeal flora in Control and Confirmed cases group; (B) Enrichment of different nasopharyngeal flora in the Signal transduction mechanisms pathway between Control and Confirmed cases group, where blue bars in panel A indicate significantly enriched pathways in the Control group, and orange bars indicate significantly enriched pathways in the Confirmed cases group. Control group (n=9), Confirmed cases group (n=12).

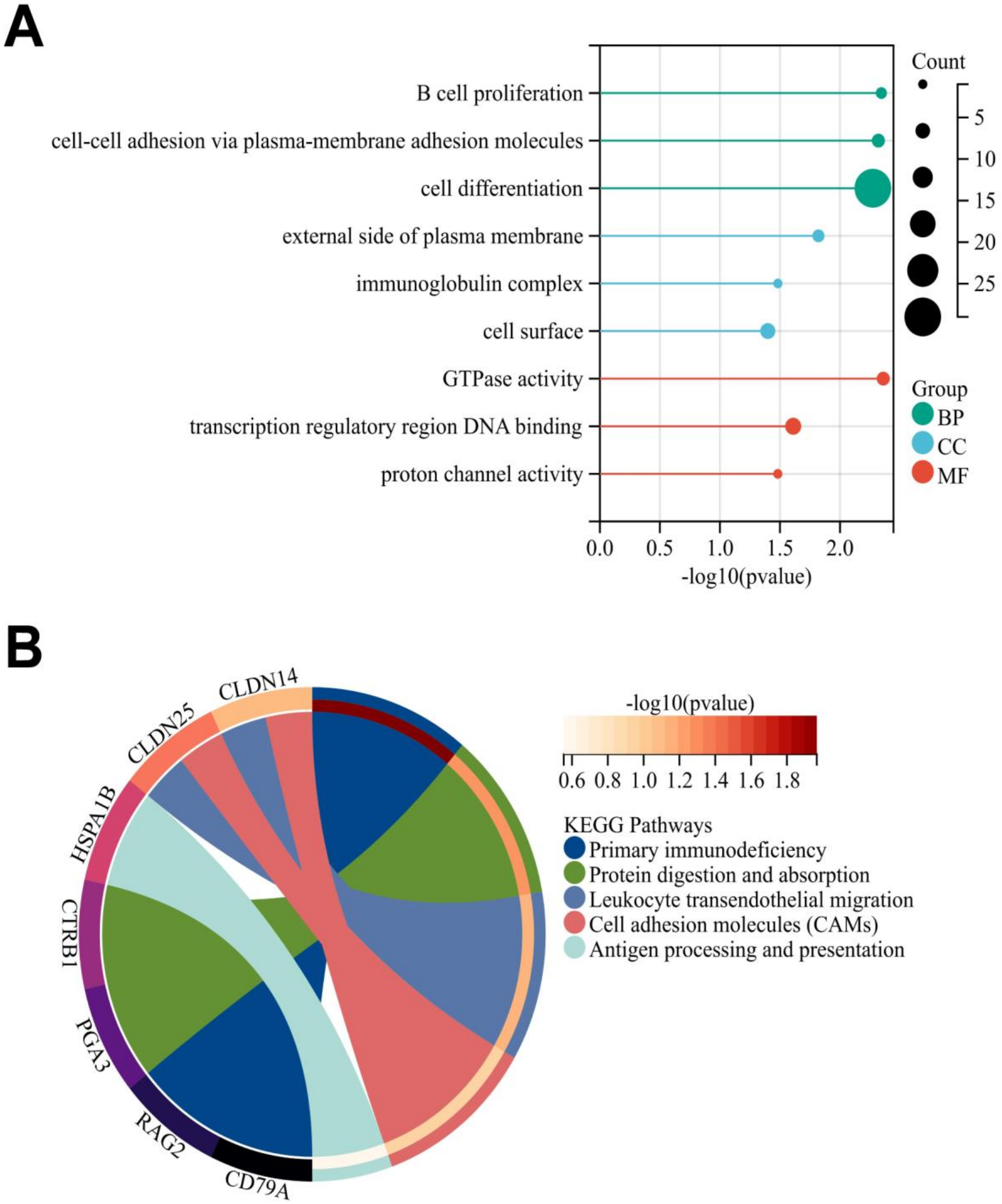

Figure S5. Enrichment analysis results of DEGs.

Note: (A) Bar graph of GO enrichment for DEGs, with green, blue, and red representing Biological Process (BP), Cellular Component (CC), and Molecular Function (MF) categories, respectively; (B) KEGG pathway enrichment plot for DEGs.

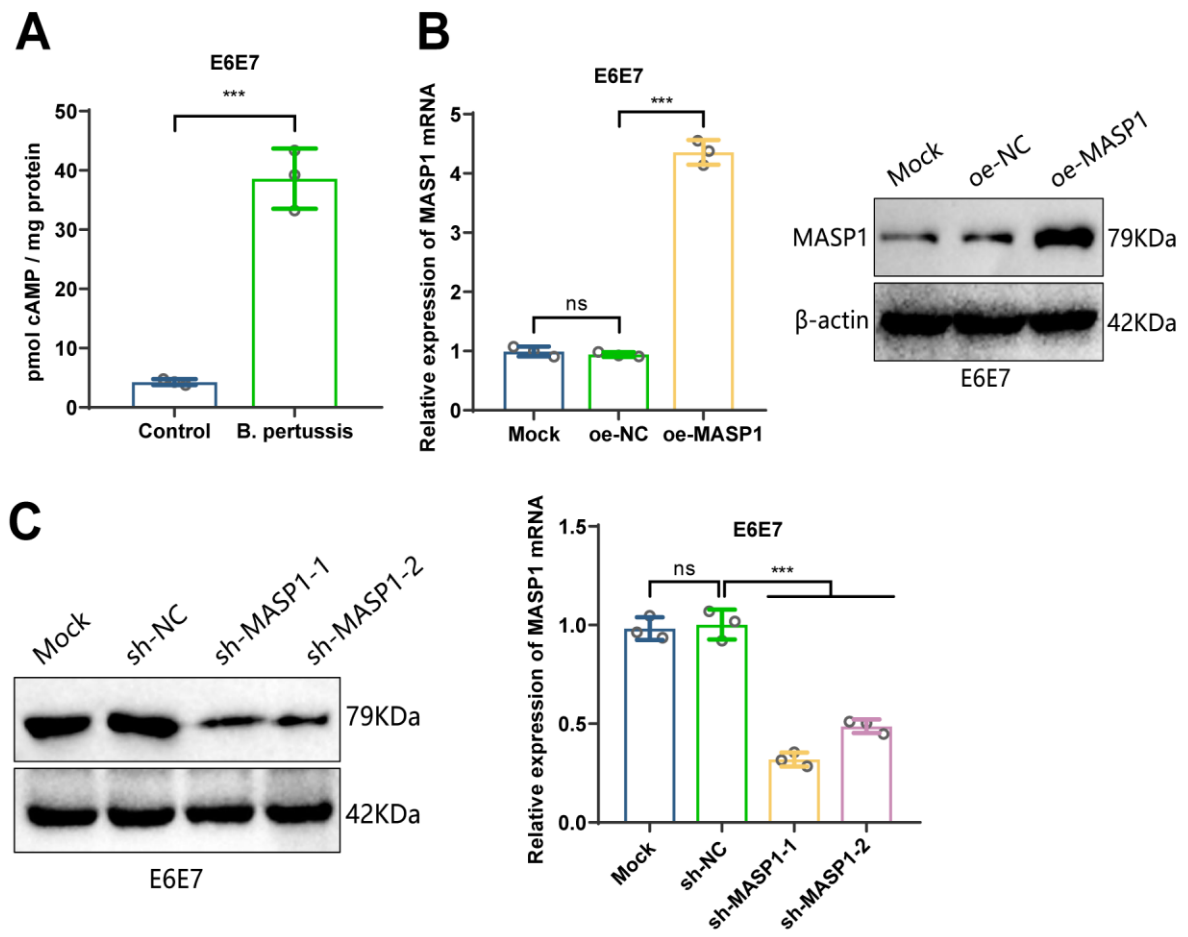

Figure S6. cAMP detection in cell infection model and validation of cell overexpression and knockdown.

Note: (A) Changes in cAMP levels in HBE135-E6E7 cells at 24 h post-B. pertussis infection; (B-C) Detection of MASP1 overexpression and knockdown in HBE135-E6E7 cells by RT-qPCR and Western blot, where dual shRNA was used for knockdown, and the one demonstrating better knockdown efficiency was selected for subsequent experiments and denoted as sh-MASP1; $^{ns}P > 0.05$, $^{***}P < 0.001$; Cell experiments were performed in triplicate.

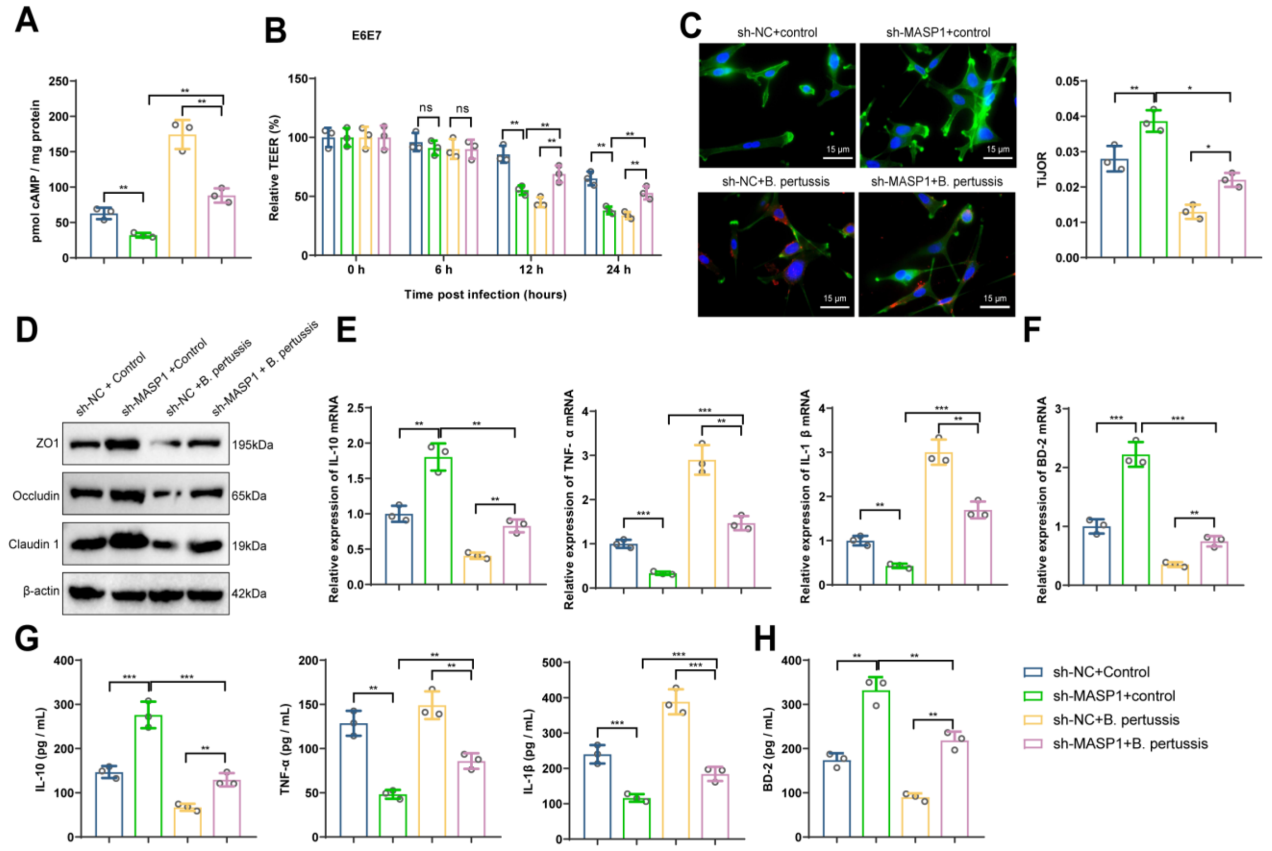

Figure S7. Impact of MASP1 knockdown on B. pertussis-induced HBE135-E6E7 cell damage.

Note: (A) Measurement of cAMP levels in sh-NC + Control, sh- MASP1 + Control, sh-NC + B. pertussis, and sh-MASP1 + B. pertussis groups; (B) TEER percentage in each group at different time points (0, 6, 12, and 24 h); (C) Representative immunofluorescence images detecting tight junction integrity (left) and statistical analysis of tight junction tissue integrity (right) (scale bar = 15 μm); (D) Expression of tight junction-associated markers in each group detected by Western blot; (E-H) Expression and secretion of inflammatory cytokines and antimicrobial peptide BD-2 in each group measured by RT-qPCR and ELISA; $^{ns}P > 0.05$, $^{*}P < 0.05$, $^{**}P < 0.01$. Cell experiments were replicated three times.

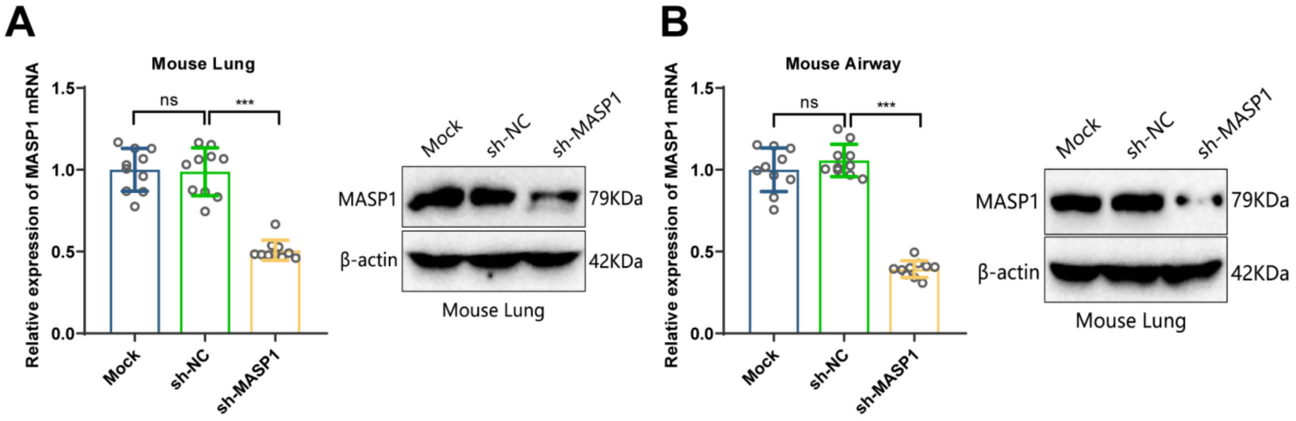

Figure S8. Validation of MASP1 knockdown in mouse lung and airway tissues.

Note: (A-B) Detection of MASP1 knockdown in mouse lung and airway tissues by RT-qPCR and Western blot; n = 10; $^{ns}P > 0.05$, $^{**}P < 0.01$.
